# Review on Toxic Effects of Di(2-ethylhexyl) Phthalate on Zebrafish Embryos

**DOI:** 10.3390/toxics9080193

**Published:** 2021-08-21

**Authors:** Wing Sum Kwan, Vellaisamy A. L. Roy, Kwan Ngok Yu

**Affiliations:** 1 Department of Physics, City University of Hong Kong, Tat Chee Ave., Kowloon Tong, Kowloon, Hong Kong, China; wingskwan8@cityu.edu.hk; 2 Department of Materials Science and Engineering, City University of Hong Kong, Tat Chee Ave., Kowloon Tong, Kowloon, Hong Kong, China; 3 James Watt School of Engineering, University of Glasgow, Glasgow G12 8QQ, UK; 4 State Key Laboratory in Marine Pollution, City University of Hong Kong, Tat Chee Ave., Kowloon Tong, Kowloon, Hong Kong, China

**Keywords:** phthalate, di(2-ethylhexyl) phthalate (DEHP), mono-2-ethylhexyl phthalate (MEHP), zebrafish embryo, toxic effect

## Abstract

Di(2-ethylhexyl) phthalate (DEHP) is widely used as a plasticizer in consumer products. People are continuously exposed to DEHP through ingestion, inhalation and dermal absorption. From epidemiological studies, DEHP has been shown to associate with various adverse health effects, such as reproductive abnormalities and metabolic diseases. Health concerns have been raised regarding DEHP exposures; therefore, relevant risk assessment has become necessary through toxicological testing of DEHP. In the past 10 years, an increasing number of DEHP toxicity studies have been using zebrafish embryos as an in vivo model due to their high fecundity, rapid embryonic development as well as optical transparency, which have now been established as an alternative of the more conventional rodent model. The aim of the present paper is to review the effects of acute (from embryo stage to ≤1 week) and chronic (from embryo stage to >1 week) DEHP exposures on zebrafish, which start from the embryonic stage, and to analyze acute and potential long-term effects induced by acute exposure and effects induced by chronic exposure of DEHP upon subjecting to exposures, starting from the embryonic stage to different developmental stages, with a view to facilitate risk assessments on DEHP exposures.

## 1. Introduction

Phthalates have been widely applied as plasticizers to enhance the flexibility of plastic in consumer products, such as toys, food containers, cosmetics, personal care products and furniture as well as medical devices [1], but at the same time have been recognized as endocrine disrupting chemicals (EDCs) [2]. Nowadays, people are pervasively exposed to phthalates through ingestion, inhalation and dermal absorption [1] since phthalates can easily migrate into the environment with time and use as they are not covalently bound to plastics [3,4,5].

**Figure 1:**
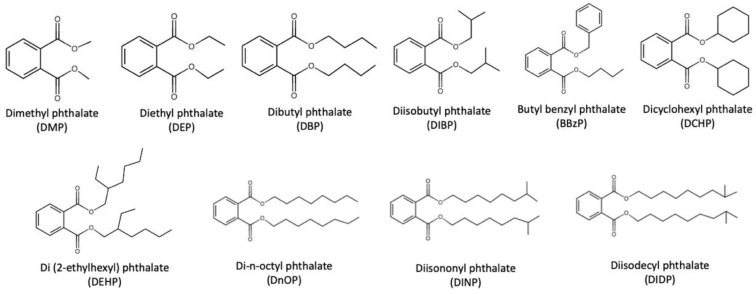
Ten widely used phthalates as summarized by Wang et al. [6].

Ten widely used phthalates were summarized by Wang et al., including dimethyl phthalate (DMP), diethyl phthalate (DEP), dibutyl phthalate (DBP), diisobutyl phthalate (DIBP), butyl benzyl phthalate (BBzP), dicyclohexyl phthalate (DCHP), di(2-ethylhexyl) phthalate (DEHP), di-n-octyl phthalate (DnOP), diisononyl phthalate (DINP) and diisodecyl phthalate (DIDP), the structures of which are depicted in Figure 1 [6].

In general, phthalates can be categorized as lower molecular weight (LMW) phthalates (e.g., DMP, DEP, BBzP) and higher molecular weight (HMW) phthalates (e.g., DEHP, DINP, DIDP) [7]. LMW-phthalates are used in paints, solvents, adhesives and body-care products, while HMW-phthalates are widely used as plasticizers. Notably, DEHP, which is the most extensively used phthalate accounting for nearly 50% of global phthalate consumption, has been associated with various adverse health effects [8]. In particular, DEHP has been classified as possibly carcinogenic to humans (Group 2B) by the International Agency for Research on Cancer Working Group [9] and also classified as priority environmental pollutants according to the United States Environmental Protection Agency (EPA) [10]. Furthermore, DEHP is restricted in children’s toys in the European Union [11], the United States [12] and Canada [13].

**Figure 2:**
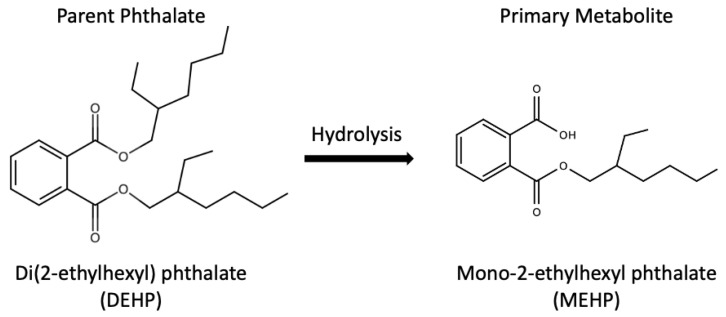
Metabolic pathway from DEHP to MEHP [6].

In relation, mono-2-ethylhexyl phthalate (MEHP), the major metabolite of DEHP, was used as a biomarker of DEHP exposure, thanks to the metabolic pathway from DEHP to MEHP as shown in Figure 2 [6]. MEHP was detected in human urine, breast milk, blood and serum [14,15,16]. Besides, DEHP has been associated with various adverse health effects, such as reproductive abnormalities, metabolic diseases as well as allergy and asthma from epidemiological studies [17,18,19,20,21,22]. Zarenan et al. reviewed adverse health effects induced by DEHP in terms of effects on reproductive system, pregnancy outcome and respiratory health, as well as carcinogenesis in several in vitro, in vivo and epidemiological studies [8]. Furthermore, prenatal and perinatal DEHP exposures have been linked with restricted growth, delayed lung maturation, neuronal, behavioral and reproductive toxicity as well as obesity in rats [23,24,25,26,27]. Notably, in the past 10 years, an increasing number of DEHP developmental toxicity studies were carried out using zebrafish (*Danio rerio*) embryos as an in vivo model, which was considered an alternative to the conventional rodent model.

Zebrafish has become a popular vertebrate model widely applied in toxicological studies due to its high fecundity, rapid embryonic development as well as optical transparency [28,29]. Moreover, development of zebrafish and mammals are similar. Importantly, the genomes of zebrafish and human share approximately 70% homology, which has made zebrafish a desirable model for studying human diseases [30]. Segner reviewed the studies which employed the zebrafish model to evaluate endocrine disrupting effects of different EDCs, such as Bisphenol A (BPA), ethynylestradiol (EE2) and phytosterols [31]. Besides, researchers adopted zebrafish embryos for studying developmental toxic effects of EDC exposures [32,33,34].

**Figure 3:**
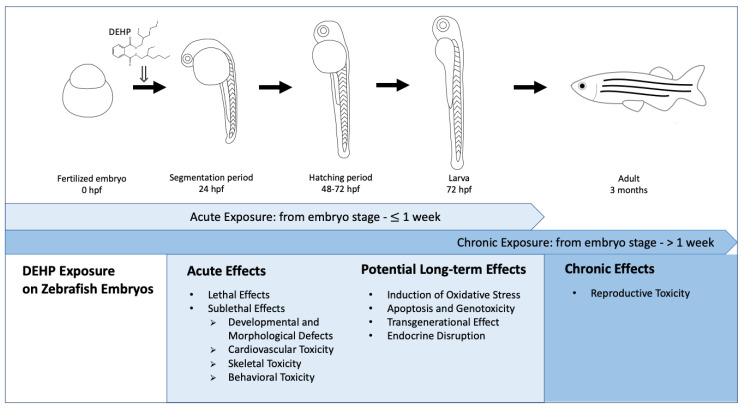
Effects of acute (from embryo stage to ≤ 1 week) and chronic (from embryo stage to > 1 week) DEHP exposures on zebrafish, which started from the embryonic stage to different developmental stages of the zebrafish [35]. (hpf: hours post-fertilization).

In the present review, a literature search was performed through Google Scholar (https://scholar.google.com/, accessed on 26 January 2021 (DEHP) and 17 March 2021 (MEHP)) with the keywords “DEHP AND zebrafish embryo” and “DEHP AND zebrafish larvae” for DEHP exposures as well as the keywords “MEHP AND zebrafish embryo” and “MEHP AND zebrafish larvae” for MEHP exposures. In particular, this review only included effects of DEHP or MEHP exposures on zebrafish, which started from the embryonic stage. In other words, studies that started to treat zebrafish with DEHP or MEHP at later developmental stages (i.e., larval, juvenile and adult stages) were excluded. Moreover, in the present review, the term “acute exposure” referred to exposures which started from the embryo stage to 1 week, while the term “chronic exposure” referred to exposures which started from the embryo stage and assessment of the zebrafish at an age more than 1 week. We summarized the effects of acute and chronic DEHP exposures on zebrafish, which started from the embryonic stage, and analyzed acute and potential long-term effects induced by acute exposures, and effects induced by chronic exposures of DEHP through various biological endpoints at different developmental stages of the zebrafish, in order to facilitate risk assessment on DEHP, as outlined in Figure 3.

## 2. Acute Effects Induced by Acute Exposure

### 2.1. Lethal Effects

Standardized endpoints have been established for evaluating the mortality of zebrafish embryos in toxicity tests. Lethal endpoints included coagulation of eggs or embryos, non-detachment of tail, somite development failure as well as lack of heartbeat [36]. Increased mortality rate was reported in DEHP-exposed zebrafish embryos [37,38,39]. In particular, Muhammad et al. found a mortality rate of 37.6% for zebrafish embryos at 72 h post-fertilization (hpf) upon exposures to 0.5 µg/L DEHP [39]. Two separate studies made use of the mortality rate at 168 hpf as the endpoint in order to compare the toxic effects on zebrafish embryos among 6 types of phthalates, including DEP, DBP, DMP, BBzP, DnOP and DEHP [37,38]. When exposed to 50 µg/L phthalate, the highest mortality rate (19.5%) was found in DEHP-treated embryos among all phthalate-treated embryos [38]. However, when a higher phthalate concentration (10 mg/L) was used, the mortality rate of DEHP ranked 5th among those tested phthalates [37]. The lethal concentration that caused 50% mortality (LC50) of DEHP on zebrafish embryos was determined to be 2.5 µg/L in zebrafish embryos at 72 hpf [40]. An independent study reported the LC50 value of DEHP for zebrafish embryos from 72 to 168 hpf as 54.02 mg/L [41]. Regarding MEHP exposures, a significant increase in the mortality rate was observed in MEHP-exposed embryos in a dose- and time-dependent manner [42]. Table 1 summarizes the lethal effects from acute exposures to DEHP or MEHP on zebrafish embryos.

### 2.2. Sublethal Effects

#### 2.2.1. Developmental and Morphological Defects

To assess sublethal effects of a toxic substance, development toxicity and teratogenic toxicity in zebrafish embryos were employed. Common morphological abnormalities included altered hatching rate, altered body size, body weight and heart rate, body curvature, induction of yolk sac and pericardial edema, spontaneous movement inhibition, malformation in swim bladder as well as pigmentation [35,43].

An increased deformity rate was reported in DEHP-exposed zebrafish embryos [37,38,41]. The deformity rate reached 20% in zebrafish embryos treated with 10 mg/L DEHP, where swimming-bladder inflation was the most commonly observed defect [41]. Pu et al. and Hamid et al. coincidently reported bent spine as one of the discernable phenotypes [37,38]. DEHP treatment with 2.5 µg/L significantly reduced the hatching rate of zebrafish embryos at 48 and 72 hpf compared to the control [44]. In a separate study, a hatching rate 62.4% was reported in the DEHP-treated group at 72 hpf, with the concentration as low as 0.5 µg/L [39]. On the other hand, body length reduction was reported in DEHP-exposed zebrafish embryos [44,45,46,47]. Tran et al. reported that the body lengths of 120 hpf zebrafish embryos were decreased upon DEHP exposures to the highest tested concentration (100 mg/L), which were however not significantly different after DEHP exposures to lower concentrations (0.5 µg/L–10 mg/L) [46]. Kinch et al. demonstrated that DEHP exposures induced a more serious effect on the morphology at earlier time points (3–24 hpf and 3–48 hpf) than at later time points (3–72 hpf) [45]. Exposures from 3 to 24 hpf to 5.2 nM DEHP significantly reduced the body length, tail length as well as yolk extension length of zebrafish embryos compared to the control, while no effects were observed with DEHP exposures at 3–48 hpf and 3–72 hpf [45]. Opposite results were obtained by Ustundag et al. and Mu et al. in that DEHP could cause yolk sac edema with longer treatment durations [44,48]. Mu et al. found that 250 µg/L of DEHP significantly induced yolk sac edema in 48 hpf embryos as well as increased ratios of yolk sac area to fish body area in DEHP-dosed embryos when compared with control embryos [48]. In a separate study, Mu et al. reported that exposure to as low as 2.5 µg/L of DEHP could cause yolk sac edema in 72 hpf embryos [44]. Kinch et al. found that the forebrain lengths of embryos treated with DEHP from 3 to 48 hpf were significantly reduced by 10% when compared to the negative controls [45]. In the same study, upon exposure to DEHP or thyroid hormone, the head length was decreased by 20% relative to the controls when exposed from 3 to 24 hpf. The results suggested that the head morphology might be changed through interactions with thyroid hormone receptors [45]. Hyperemia and dark pigmentation were also detected in DEHP-exposed zebrafish embryos [41]. For MEHP exposures, Park et at. reported effective concentrations (EC) based on biological responses (mortality and abnormality) of zebrafish embryos after exposures to eight different concentrations of MEHP (namely, 0, 0.1, 1.6, 3.1, 6.3, 12.5, 25 and 50 µg/mL) for 6 days [49]. No observed effect concentration (NOEC), EC10, EC50 and EC100 were determined as <6.09, 9.77, 29.98 and 50 µg/mL, respectively [49]. Kamstra et al. reported reduction in body length [50], while Sant et al. found abnormalities in the swim bladder in MEHP-exposed larvae [51]. On the other hand, Lu et al. revealed decreases in the hatching rate, heart rate and body length as well as increased deformity in MEHP-exposed embryos when compared to the controls [42]. Table 2 summarizes developmental and morphological defects from acute exposures to DEHP or MEHP in zebrafish embryos.

#### 2.2.2. Cardiovascular Toxicity

Studies report that DEHP could impose cardiovascular toxicity to zebrafish embryos. Treatment with 250 µg/L DEHP induced intense apoptotic signals in the heart region in zebrafish embryos at 72 hpf relative to the control [52]. On the other hand, DEHP was reported to induce pericardial edema in zebrafish embryos [41,44,52]. Pericardial edema was observed at 72 or 96 hpf after treatment with DEHP in the µg/L range [44,52]. Exposure to a higher DEHP concentration (10 mg/L) from 72 to 168 hpf also led to the same effect [41]. However, effects on the heart rate induced by DEHP were different when lower (in the µg/L range) or higher (in the mg/L range) doses were used. The heart rate in zebrafish embryos has been widely used as an endpoint for assessing heart toxicity. Pu et al. and Lu et al. demonstrated that DEHP doses in the mg/L range could reduce the heart rate in embryos at 3 days post-fertilization (dpf) [37,42]. In contrast, the heart rate was significantly increased in embryos at a later development stage (168 hpf) upon treatment with lower DEHP doses (25 and 50 µg/L) [38]. For MEHP exposure, Lu et al. reported reduction of heart rate in embryos upon treatment with MEHP in the mg/L range [42]. Table 3 summarizes the cardiovascular toxicity from acute exposures to DEHP or MEHP on zebrafish embryos.

#### 2.2.3. Skeletal Toxicity

DEHP has been associated with skeletal and spinal abnormalities [37,44]. Exposures to DEHP from 4 to 72 hpf could instigate axial curvature in zebrafish embryos [44]. Bent spinal curvature as well as spinal dysplasia were also shown in DEHP-exposed zebrafish embryos at 168 hpf [37]. Table 4 summarizes skeletal toxicity from acute exposure to DEHP on zebrafish embryos.

#### 2.2.4. Behavioral Toxicity

Exposures to DEHP have exerted behavioral toxicities on zebrafish embryos [37,42,46,53]. Spontaneous movement inhibition has been reported in zebrafish embryos upon DEHP exposures [37,53]. Spontaneous movement was established as an important marker to assess the developmental status and behavioral ability of zebrafish embryos, which was initiated by the spinal cord at approximately 24 hpf and was suggested to associate with hatching [43]. In one of the related studies, zebrafish embryos were exposed to seven concentrations of DEHP (0, 10, 50, 90, 120, 150, 200 mg/L), and the automatic movement number in 20 s of the embryos was assessed using a stereomicroscope at 24 hpf. The results showed that DEHP significantly inhibited movement at higher concentrations (90–200 mg/L) [37]. Similar results were found by Qian et al. who reported an inhibitory effect on the movement of zebrafish embryos upon DEHP exposures as low as 50 µg/L [53]. Locomotor activity was another marker to assess the behavioral ability in zebrafish embryos. DEHP exposure was confirmed to alter locomotor activities in zebrafish embryos [42,46]. In a related study, zebrafish embryos were exposed to four concentrations of DEHP (0, 10, 25, 50 µM) at 4 hpf, and after the 96 h exposure time point, the swimming activities were recorded using the Zebralab Video-Track system (ViewPoint Life Science, France) [42]. The results indicated that an exposure to 50 µM DEHP could significantly inhibit the locomotor activity compared to the control [42]. In a separate study, the locomotor activities under alternating light–dark conditions of DEHP-treated zebrafish embryos at 120 hpf were evaluated via the ZebraBox tracking system (ViewPoint Life Science, France) [46]. Suppression of locomotor activities was reported in DEHP-exposed embryos under the dark condition, while stimulation of locomotor activities was found under the light condition [46]. Together, the lowest effective concentration of DEHP in inhibition of locomotor activity of zebrafish embryos was 5 µg/L as reported by Tran et al. [46]. As mentioned in Section 2.2.1, morphological developmental effects were induced upon DEHP exposures to as low as 2.0 µg/L (5.2 nM), with the observable body length, tail length, yolk sac extension length, forebrain length and head width decreased as reported by Kinch et al. [45]. From available data, the dose of DEHP, which triggered morphological effects, was lower than that which inhibited locomotor activity in zebrafish embryos. No existing data showed effect concentrations in locomotor activity at exposure levels where no morphological or developmental abnormalities were observed. More studies are required for understanding the relationship between morphological and developmental aberrations and behavioral toxicity in zebrafish embryos. Table 5 summarizes the behavioral toxicity from acute exposures to DEHP or MEHP on zebrafish embryos.

### 2.3. Regulation of Genes and Possible Mechanisms

In Section 2.2, we summarized the sublethal effects induced by DEHP or MEHP in terms of developmental and morphological defects, cardiovascular, skeletal and behavioral effects. The mechanisms behind DEHP- or MEHP-induced effects on zebrafish embryos are important for understnading the mode of action. DEHP was reported to change gene expression in zebrafish embryos, which was revealed using different techniques, including immunohistochemical staining and reverse transcription polymerase chain reaction (RT-PCR) analyses. Ustundag et al. studied the effect on gene expression upon the exposure of 2.5 µg/L of DEHP to zebrafish embryos from 4 to 48 hpf. Immunohistochemical staining was applied to examine the expressions PCNA, Wnt3a and *β*-catenin genes in zebrafish embryos [44]. Intense staining of PCNA, Wnt3a and *β*-catenin were observed in the DEHP group compared to the weak staining shown in the control group. The authors further conducted reverse transcription polymerase chain reaction (RT-PCR) analysis to examine gene expressions in 72 hpf zebrafish embryos. Expression of gsk3*β* was significantly increased in the DEHP group relative to the control [44]. Studies also reported that DEHP altered gene expressions related to the skeletal development pathway in 96 hpf zebrafish embryos [37,53]. Exposure of environmentally relevant concentrations of DEHP at 50 µg/L significantly upregulated the expressions of genes in 96 hpf zebrafish embryos involved in skeletal development in terms of skeleton (*spp1*), notochord (*ngs*, *col8a1a*) and muscle (*klhl41a*, *smyd2b*, *stac3*) [53]. However, the expression of *col8a1a* was significantly reduced by DEHP at 250 µg/L. Together, the results demonstrated that DEHP could lead to abnormal development of the spine and skeletal system and the authors proposed that different transcriptional effects could be induced under different concentrations [53]. Pu et al. also reported that a higher concentration (200 mg/L) of DEHP activated the expression levels of four major skeletal-related genes (*sp7*, *runx2b*, *gpc4a*, *shha*) in zebrafish embryos [37].

On the other hand, multi-omics approaches were also employed to investigate the mechanisms of DEHP-induced developmental toxicities [48,52]. Omics approaches in biology, including genomics, epigenomics, transcriptomics, proteomics, lipidomics and metabolomics, can reveal alterations in different domains upon toxicant exposures and provide insights into possible mechanisms as well as potential toxic effects and diseases [54]. Mu et al. examined the effects on zebrafish embryos upon exposures to 50 µg/L of DEHP through transcriptomic, proteomic and lipidomic approaches [48]. From transcriptomic profiling analysis, significant alteration was observed in expression of transcripts related to different biological processes involved in lipid metabolism and skeletal development, e.g., steroid binding, lipid binding, lipid transport, fatty-acid metabolism, skeletal muscle tissue development, stress response as well as chemokine activity [48]. On the other hand, the data from proteomic profiling analysis suggest significant changes in the levels of 235 proteins (217 up-regulated and 18 down-regulated) [48]. The data revealed that DEHP significantly induced various processes in zebrafish embryos, including lipase activity regulation, lipid absorption, lipid catabolism and lipid metabolism [48]. REVIGO similarity analysis was further applied, which revealed that lipid digestion, lipid metabolism, lipid transport and lipase activity regulation were the major pathways activated by DEHP exposures [48]. From lipidomic profiling analysis, among 207 identified lipid species in zebrafish embryos, DEHP was shown to significantly lower the levels of neutral glycosphingolipids (Creg1), cholesterol esters (Che), diglycerides (DG), triglycerides (TG) and fatty acids (FA) [48]. The study also reported that DEHP altered the expression of protein levels involved in immune responses (TNF*α*, NF-k*β*, il-1*β*, il-8, tp63) [48]. Based on these findings, the authors inferred that DEHP-induced immune response might be a result of disruption of lipid homeostasis [48]. In a separate study, Mu et al. further investigated possible developmental pathways upon exposures to 50 µg/L of DEHP via transcriptomic and DNA methylation profile analysis, particularly the genes involved in heart development [52]. From transcriptomic analyses, among 32,477 genes in the reference genome of zebrafish, the research group reported that 371 transcripts (234 up-regulated and 137 down-regulated) were significantly altered in embryos after their exposure to 50 µg/L DEHP, followed by the gene ontology analysis. [52]. The results showed that exposure to 50 µg/L of DEHP significantly induced pathways of steroid binding (*paqr5b*, *nr1h4*, *fabp10a*), cyclase activator activity (*guca1a*, *guca1d*), actomyosin structure organization (*klhl41a*, *csrp3*, *cnn1b*, *ctnt*), chemokine receptor binding (*ccl27a*, *ccl39.3*), myofibril assembly (*csrp3*, *klhl41a*, *ctnt*), notochord development (*col8ala*, *ngs*, *fbn2b*), skeletal system development (*spp1*), heart contraction (*smyd2b*, *csrp3*) as well as heart process (*smyd2b*, *csrp3*), which were related to heart development [52]. Differential gene transcriptions, including *nppa*, *My17*, *Tbx5b*, *smyd2b*, *klhl41a*, *ctnt* and *cmlc1* were altered in embryos after DEHP exposures, which were further confirmed using quantitative polymerase chain reaction (qPCR) [52]. In the same study, the global change of DNA methylation in zebrafish embryos was assessed using methylated DNA immunoprecipitation sequencing (MeDIP-Seq). Mu et al. also reported significant hypomethylation of *nppa*, *ctnt* and hypermethylation of *tbx5b* in DEHP-exposed zebrafish embryos, which were related to heart development [52]. Comparisons on transcriptomic and DNA methylation profiles suggested that transcriptional alteration of gene might be associated with modified DNA methylation [52].

Junaid et al. studied the toxicity in zebrafish embryos induced by acute exposures to DEHP at environmentally relevant concentrations (0–400 µg/L) [55]. The results showed that 400 µg/L of DEHP could activate the PI3K-AKT-mTOR pathway in zebrafish embryos as evidenced by significant upregulation of the transcripts of key genes involved in the pathway (*pik3r1*, *akt1*, *mtor*, *ps6kb*) [55]. Junaid et al. further used the cyp1a transgenic zebrafish to monitor the effect of DEHP exposure on the AhR activity. Concentration-dependent increases in the levels of fluorescent signals in DEHP-exposed *Tg(cyp1a:gfp)* zebrafish embryos were observed, which indicated that short-term low-concentration exposures to DEHP could induce AhR activity in the embryos [55]. Do et al. also employed transgenic zebrafish as the model particularly to explore possible mechanisms of DEHP-induced abnormal neurobehavior. After confirming the inhibition of locomotor activities in DEHP-exposed zebrafish embryos, as described in Section 2.2.4., the authors further found significant reduction in fluorescent intensity in the transgenic zebrafish *Tg (HuC:eGFP)* upon DEHP exposures [46]. The study further reported the potential of DEHP in interfering with neurotransmission as evidenced through upregulation of *ache* and down regulation of *th* gene expression, which were involved in the dopamine system [46].

Regarding MEHP exposures, Sant et al. observed that exposures to 200 µg/L of MEHP significantly enhanced vacuolization and types of vacuolization in the livers of 96 hpf zebrafish larvae through histological analysis [51]. Furthermore, the zebrafish larvae were exposed to 200 µg/L of MEHP from 6 to 120 hpf and were assessed on the 15th day through different endpoints. The lipid contents in the zebrafish larvae were assessed via red O staining, which revealed that MEHP exposures significantly increased lipid accumulation in the livers and brains in the larvae compared to the control group [51]. Expression of peroxisome proliferator-activated receptor (PPAR) alpha target *fabp1a1* was also found to have increased in MEHP-exposed larvae [51]. A parallel experiment was also conducted to investigate the involvement of nuclear factor erythroid 2-related factor 2 (Nrf2) signaling in MEHP-induced larval steatosis using the nrf2a mutant zebrafish. However, the results demonstrated that MEHP-induced larval steatosis was independent of Nrf2 signaling [51]. Table 6 summarizes the regulation of genes and possible mechanisms from acute exposures to DEHP or MEHP on zebrafish embryos.

## 3. Potential Long-Term Effects Induced by Acute Exposure

In addition to acute developmental and teratogenic toxicity in zebrafish embryos, assessments through various biological endpoints also revealed long-term effects. Examples of established biomarkers in the fish model included immune modulation, endocrine disruption as well as genotoxicity [56]. In this section, we reviewed potential long-term effects induced by DEHP exposure in zebrafish embryos in terms of oxidative stress, apoptosis and genotoxicity, transgenic effect as well as endocrine disruption.

### 3.1. Induction of Oxidative Stress

It was well established that oxidant–antioxidant balance played a critical role in organisms to maintain cellular development growth and survival. DEHP was found to upset the oxidant–antioxidant balance in zebrafish embryos [38,40,42]. Lu et al. reported the increased ROS generation and decreased superoxide dismutase (SOD) activity in 28 hpf embryos after DEHP and MEHP exposures [42]. In a separate study, enhanced expression of oxidative stress-related genes (CAT, CuSOD, MnSOD) were reported in zebrafish embryos upon exposures to 50 µg/L of DEHP [38]. Upset of oxidant–antioxidant balance in embryos was also reported in DEHP-exposed zebrafish embryos in terms of increased lipid peroxidation (LPO) and decreased glutathione-S-transferase (GST) levels exposed to as low as 2.5 µg/L of DEHP [40]. Table 7 summarizes the induction of oxidative stress from acute exposures to DEHP or MEHP on zebrafish embryos.

### 3.2. Apoptosis and Genotoxicity

Genotoxic effects of DEHP were confirmed in various in vitro and in vivo studies and was reviewed by Caldwell, including induction of DNA damages, abnormal regulation of mitotic rate, apoptosis and cell prolifreation as well as activation of different nuclear receptors, which could contribute to cancer progression [57]. DNA damages could be induced by direct action of parental compounds of environmental contaminants or their metabolites or through indirect action by the generated reactive oxygen species (ROS) [58]. Embryos were exposed to five sublethal concentrations of DEHP (namely, 0, 0.5, 1, 5 and 10 mg/L) from 72 to 168 hpf, and DNA damages in cells of the embryos were then analyzed using comet assay [41]. The results demonstrated that DEHP significantly induced DNA strand breaks in embryos in a concentration dependent manner, when compared to controls. Boran et al. further reported upregulation of the tumor suppressor gene *p53* as well as DNA repair genes *rad51* and *xrcc5* in DEHP-exposed embryos [41]. Lu et al. further studied the involvement of the BER pathway in DEHP- and MEHP-treated zebrafish embryos through measuring the mRNA levels of genes related to the BER pathway [42]. The results showed that DEHP exposures significantly increased mRNA levels of *ogg1*, *parp1*, *pcna*, *fen1* and *lig1*, while MEHP exposures significantly increased mRNA levels of *ogg1*, *nthl1*, *apex1*, *aprp1*, *xrcc1*, *lig3*, *ung*, *pcna*, *fen1* and *lig1*, which suggested that the BER pathway played critical roles in DEHP- and MEHP-induced oxidative stress through repairing oxidative DNA damages [42].

Besides, DEHP was confirmed to induce apoptosis and alter apoptosis-related genes *(cas8*, *cas9*, *pf3*, *bcl*, *bax*) in zebrafish embryos [38,42,52]. Acridine orange (AO) staining was established as a widely used technique to study apoptosis in zebrafish embryos. Various studies used AO staining to investigate DEHP-induced apoptosis in zebrafish embryos [38,52]. Exposures to 250 µg/L of DEHP from 1.5 to 72 hpf induced apoptotic signals in zebrafish embryos, and intense apoptotic signals were observed in the heart region [52]. Hamid et al. also observed an elevated number of apoptotic cells in DEHP-treated zebrafish embryos with a lower DEHP concentration (50 µg/L) and for a longer exposure (3–96 hpf) [38]. In the same study, increased mRNA levels of the apoptosis-related genes *cas8*, *cas9*, *pf3* and *bax* were detected in DEHP-treated embryos [38]. In a separate study, mRNA expressions of *bcl2* and *bax* were found to increase in 28 hpf embryos upon exposures to higher concentrations of DEHP (25–50 µM) [42]. The mRNA levels of *bcl2* and *bax* in zebrafish embryos were also significantly altered after MEHP exposures [42]. Ustundag et al. investigated the relationship between oxidant–antioxidant balance and expression of *c-myc*, which was the gene that regulated cell proliferation and apoptosis. Increased expression of *c-myc* in DEHP-exposed embryos was reported, which was thus proposed as a protective mechanism to preserve oxidant–antioxidant balance in zebrafish embryos [40]. Table 8 summarizes the induction of apoptosis and genotoxicity from acute exposures to DEHP or MEHP in zebrafish embryos.

### 3.3. Transgenerational Effect

Kamstra et al. investigated the transgenerational effect of MEHP exposures among three generations of zebrafish larvae (denoted as F0, F1 and F2), with only the F0 generation having been exposed to 30 µM of MEHP from 0 to 6 dpf [50]. Body length differences were reported in F0 larvae at 3 and 6 dpf, which had been exposed to MEHP, while no effect was reported in F1 and F2 larvae [50]. Altered *dnmt* gene expressions (*dnmt1*, *dnmt3aa*, *dnmt3ab*, *dnmt3bb.1*, *dnmt3bb.2*) and decreased global hmC levels were reported in F0 larvae at 6 dpf. Furthermore, global demethylation in livers of F0 adult female zebrafish and reduction of global hmC levels in brain tissues of F0 adult male zebrafish were detected [50]. In the same study, MEHP exposure was found to lead to a significant increase in the average methylation at conserved non-genic elements in zebrafish (zfCNEs) when compared to the control [50]. The pathways involved in adipogenesis were enriched after MEHP exposures, which were suggested to link to a potential obesogenic effect of MHEP [50]. Transgenerational effects were further assessed through locus-specific methylation analysis by comparing differences of the methylation pattern among the three generations (F0 at 6 dpf, F1 at 6 dpf, F2 at 6 dpf, F0 at 15 dpf and F0 adult sperm). Transgenerational effects were shown at the *cbfa2t2* locus and specific CpG sites [50]. Table 9 summarizes the transgenerational effect from acute exposure to MEHP on zebrafish embryos.

### 3.4. Endocrine Disruption

DEHP was reported to cause endocrine disruptions in zebrafish embryos [38,47,48,55,59,60]. Jia et al. demonstrated that DEHP exposures disrupted homeostasis of thyroid hormones (THs) and altered expression of critical genes related to hypothalamus–pituitary–thyroid (HPT) axis in the zebrafish [59]. In that study, zebrafish embryos were exposed to five concentrations of DEHP, namely, 0, 40, 100, 200 and 400 µg/L from 2 to 168 hpf. The results revealed that whole-body thyroxinem (T4) and triiodothyronine (T3) were significantly increased after exposure to the highest tested concentration of DEHP, which subsequently led to disruption of homeostatic balance of the thyroid hormone [59]. The authors further indicated that DEHP changed the expression of thyroid stimulating hormone (*tshβ*), corticotrophin releasing hormone (*crh*), uridine diphosphate glucuronosyl transferase (*ugt1ab*), iodothyronine deiodinase (*dio2*), transthyretin (ttr) and NK2 homeobox 1 (*nkx2.1*) involved in thyroid development and thyroglobulin (*tg*) involved in thyroid synthesis [59]. Upregulation of *tshβ* and *crh* due to DEHP exposures were also observed in a dose dependent manner [59].

In a separate study, 50 µg/L of DEHP exposure was found to have significantly altered the expression of transcripts or proteins related to endocrine responses, including T3, T4, 17*β*estradiol and estrogen receptor alpha (ERα) [48]. Hamid et al. revealed that DEHP could perturb the hypothalamic–pituitary–gonadal (HPG) axis in zebrafish embryos [38], where the HPG axis was proved to be essential for normal function of the reproductive system in fish [61]. Significant upregulations of expressions in HPG-axis-pathway-related genes including *erα*, estrogen receptor beta (*erβ)*, androgen receptor (*ar)*, cytochrome P450 aromatase (*cyp19a)* and vitellogenin (*vtg*) in zebrafish embryos were observed upon exposures to 50 µg/L of DEHP. The results showed that DEHP altered *erα*, *erβ* expressions in a concentration dependent manner [38]. In a separate study, An et al. also reported significant upregulation of *erα* and *vtg* expressions in embryos upon exposures to 0.1 mg/L of DEHP; however, no significant differences were found in *erβ* expressions [60]. The study also found increased expression of *cyp19b* in DEHP-exposed zebrafish embryos [60]. In relation, Lee et al. reported that the transcript level of HPG-axis-related genes (*vtg1*, *erα*, *cyp19a1b*) were significantly altered upon DEHP exposures [47]. In another study, Junaid et al. studied cancer cell migration in DEHP-exposed zebrafish through injection of labelled breast cancer cells into 72 hpf larvae followed by two different levels of DEHP exposures (400 and 1600 µg/L) for 6 h [55]. The distances traveled by cancer cells in zebrafish were determined through the fluorescence sites and intensities. DEHP-induced cancer-cell migration was confirmed through changes in the fluorescence distribution in the yolk, gut and tail in two DEHP-dosed groups when compared to the control group, while the total fluorescence intensity was unchanged [55].

MEHP was also found to cause endocrine disruptions in zebrafish embryos [62,63,64,65]. Zhai et al. reported MEHP exposures elevated T3 and reduced T4 contents in zebrafish embryos [62]. The results linked the genes involved in thyroid hormone metabolism (iodothyronine deiodinase (*Dio2*), UDP-glucuronosyltransferases *(UGT1ab*)) to T4 content reduction. The genes related to thyroid development (*Nkx2.1*, Paired Box 8 (*Pax8*)), thyroid hormone synthesis (TSHβ, sodium/iodide symporter (NIS) and TG) and TH transport (transthyretin, TTR) were significantly altered after MEHP exposures [62]. In a separate study, MEHP was reported to disrupt glutahione (GSH) homeostasis in zebrafish embryos [63]. GSH played a critical role in embryonic development and organogenesis, which were revealed through monochlorobimane (MCB) staining [63]. In this study, the zebrafish were exposed to 200 µg/L MEHP from 3 hpf to either 48 or 72 hpf. Reductions in MCB fluorescence intensities in the body, heart, brain ventricle and somite 12 were found at both time points, while more serious disruptions were observed in 48 hpf embryos. These results confirmed that MEHP exposures disrupted GSH utilization in specific tissues during embryo development [63]. In separate studies, MEHP was found to disrupt pancreatic organogenesis in zebrafish embryos [64,65]. The endocrine tissue within the pancreas was called islet of Langerhans, which contained α-cells and β-cells responsible for secreting glucagon and insulin, respectively. Sant et al. studied the effect of exposures to 0.7 µM MEHP on the pancreatic islet development in transgenic zebrafish *Tg(ins:GFP)* embryos at 48 hpf [65], and demonstrated that MEHP produced hypomorphic islets with evidence of reduction in β-cell cluster area [65]. In relation, Jacob et al. confirmed that exposures to 200 µg/L MEHP reduced β-cell as well as α-cell cluster area in transgenic zebrafish *Tg(ins:GFP)* and *Tg(gcga:GFP*), respectively. Both these studies confirmed MEHP exposures increased the frequency of pancreatic islet morphology variants such as fragmented and ectopic islets, relative to the control [64,65]. Jacob et al. also reported that MEHP exposures altered expression of genes related to endocrine hormone (preproinsulin a (*insa)*, *somatostatin 2 (sst2*)), exocrine (pancreasspecific transcription factor 1a, *ptf1a*) and glutathione (GSH) (glutathione disulfide reductase (gsr), glutathione S-transferase pi 1 (gstp)) in wildtype zebrafish embryos. Changes in the pancreatic structure were also observed in MEHP-exposed wildtype and mutant zebrafish embryos [64]. Table 10 summarizes the endocrine disruption from acute exposures to DEHP or MEHP in zebrafish embryos.

## 4. Effects from Chronic Exposure

Chronic toxicities on the development, reproduction and intestines in zebrafish were assessed after exposures to DEHP from the embryo to adult stage [39,66]. After being exposed for 1 month to 0.5 µg/L DEHP, the mortality rate of hatchlings was reported as 60.57%, compared to the rate of 11.11% observed for the control group [39]. Muhammad et al. assessed developmental and reproductive toxicities in zebrafish upon 6-month exposures to 0.5 µg/L DEHP through the survived hatchlings at 1 month. Both the body lengths and weights of female and male zebrafish adults were significantly reduced [39]. After 6-month DEHP exposures, the gonad somatic index (GSI) was reduced for both female and male zebrafish adults, which implied interruption of gonad development. Breeding disruption in zebrafish adults was also reported in terms of decreasing egg production and increasing non-fertilization rate [39]. Muhammad et al. further applied histological examination to investigate the development of testis and ovary in zebrafish treated with 0.5 µg/L of DEHP. The testes and ovaries of zebrafish in the control group were confirmed normal. In the DEHP-dosed male group, well-developed testes were observed while disruption of tubules as well as reduction in spermatogonia and spermatocyte were found [39]. In the DEHP-dosed female group, undeveloped ovaries were identified from the peri-nucleolar oocytes and early cortical alveolar oocytes [39]. These results showed that long-term DEHP exposures posed toxicity on growth, reproduction and fecundity in zebrafish. In a separate study, a gender-based zebrafish study was conducted to investigate the effects of exposures to environmental concentrations (0, 10, 33, 100 µg/L) of DEHP in embryos to 3.5-month adults [66]. Increased body lengths and weights as well as altered intestinal microbiota and bacteria were reported in both male and female zebrafish following DEHP exposures [66]. Increased conditional factors (K) were found in female zebrafish treated with 10 µg/L of DEHP and in male zebrafish treated with 33 µg/L of DEHP. Reduction in villus width and goblet cells per villus in male and reduction of tunica muscularis thickness in female were also reported in DEHP-treated zebrafish via histological analysis [66]. Besides, in the intestines of DEHP-treated zebrafish, the content of energy metabolites (TG, PY, FA, Glu) and the expression of key genes involved in immune response (*tlr-5*, *il-1β*, *nf-kb*, *il-8*) were altered [66]. Based on these results, the study suggested that DEHP could induce obesity as evidenced from altered developmental indices, intestinal microbial community and metabolic homeostasis [66]. Table 11 summarizes the effects from chronic exposure to DEHP on zebrafish embryos.

## 5. Summary

DEHP is the most extensively applied phthalate accounting for nearly 50% of global phthalate consumption and has been associated with various adverse health effects. Health concerns have been raised regarding DEHP exposures and in consequence, risk assessment on DEHP exposures has become a required procedure. In the present review, we focused on the toxicities induced by acute or chronic DEHP exposures on zebrafish embryos with the exposure window, which started from the embryo stage. Apart from the parent phthalate DEHP, its major metabolite MEHP could also be detected in the human body and related to different adverse health effects, which was mentioned in Section 1. Both the parent phthalate, DEHP, and its major metabolite, MEHP, could induce various adverse developmental effects on zebrafish embryos upon acute or chronic exposures, which were summarized in Table 1, Table 2, Table 3, Table 4, Table 5, Table 6, Table 7, Table 8, Table 9, Table 10, Table 11, with the lowest effective dose, exposure widow and time point for assessing each of the biological endpoints in order to facilitate the comparison between the outcomes from different studies.

The linkage between various developmental and teratogenic toxicities and the underlying mechanisms requires further investigation. For comparing the toxicities of DEHP and MEHP exposures, a parallel experiment showed that the lowest effective doses of DEHP and MEHP were different to induce the reduction of survival rate, hatching rate and body length as well as an increase in deformity rate [42]. Besides, the data from a zebrafish developmental screening showed that only the active metabolite MEHP was toxic, while the parent DEHP tested negative in the concentration-response study [67]. More studies are required to investigate the lowest effective dose as well as mechanisms underlying different developmental effects of DEHP and MEHP, and to further reveal whether they pose the same toxic effects through the same mechanisms to living organisms.

As many key developmental signaling pathways and their regulations are conserved between zebrafish and mammals, the zebrafish model has been employed for investigating mammalian disease as well as developmental pathways on the molecular basis [68,69]. MEHP have been shown to induce hepatic toxicity [51], and DEHP have been reported to alter critical genes related to HPT axis and produce reproductive toxicity on zebrafish [39,58]. Since zebrafish develops relevant structures, e.g., liver for metabolic conversions [70] as well as the thyroid gland which is responsible for controlling development [71], it might facilitate assessment of toxicity in mammals. More comprehensive studies are required to provide better understanding on the mechanistic pathways of DEHP- and MEHP-induced toxic effects and to establish a correlation between zebrafish developmental toxicity and mammalian developmental toxicity that may facilitate further development on risk assessment.

## Figures and Tables

**Table 1:** Lethal effects from acute exposures to (a) DEHP and (b) MEHP on zebrafish embryos. ** The concentration was converted from nM or µM using the molar mass of MEHP 278.34 g/mol.

(a) DEHP				
Endpoint	Lowest Effective Dose	Exposure	Endpoint Time	References
mortality rate increased	0.5 µg/L	27–72 hpf	72 hpf	[39]
	25 µg/L	3–168 hpf	168 hpf	[38]
	200 mg/L	1–168 hpf	168 hpf	[37]
survival (LC50)	2.5 µg/L	start at embryo stage—72 hpf	72 hpf	[40]
	54.02 mg/L	72–168 hpf	72–168 hpf	[41]
**(b) MEHP**				
**Endpoint**	**Lowest Effective Dose**	**Exposure**	**Endpoint Time**	**References**
mortality rate increased	** 2.8 mg/L	start at embryo stage—36/48/60/72/96 hpf	** 2.8 mg/L: 60, 72, 96 hpf; ** 7.0 mg/L & ** 14 mg/L: 36, 48, 60, 72, 96 hpf	[42]

**Table 2:** Developmental and morphological defects from acute exposures to (a) DEHP and (b) MEHP in zebrafish embryos. * The concentration was converted from nM or µM using the molar mass of DEHP 390.57 g/mol. ** The concentration was converted from nM or µM using the molar mass of MEHP 278.34 g/mol.

(a) DEHP				
Endpoint	Lowest Effective Dose	Exposure	Endpoint Time	References
hatching delayed	0.5 µg/L	27–72 hpf	72 hpf	[39]
	2.5 µg/L	4–48/72 hpf	48, 72 hpf	[44]
deformity rate increased	25 µg/L	3–168 hpf	168 hpf	[38]
	200 mg/L	1–168 hpf	168 hpf	[37]
body length decreased	* 2.0 µg/L	3–24 hpf	24 hpf	[45]
	100 mg/L	2–120 hpf	120 hpf	[46]
	100 mg/L	6–168 hpf	168 hpf	[47]
tail length decreased	* 2.0 µg/L	3–24 hpf	24 hpf	[45]
swim bladder inflation inhibited	500 µg/L	72–168 hpf	168 hpf	[41]
yolk sac edema	2.5 µg/L	4–72 hpf	72 hpf	[44]
	50 µg/L	2–48 hpf	48 hpf	[48]
yolk extension length decreased	* 2.0 µg/L	3–24 hpf	24 hpf	[45]
hyperemia	10 mg/L	72–168 hpf	168 hpf	[41]
dark pigmentation	10 mg/L	72–168 hpf	168 hpf	[41]
forebrain length decreased	* 2.0 µg/L	3–48 hpf	48 hpf	[45]
head width decreased	* 2.0 µg/L	3–24 hpf	24 hpf	[45]
**(b) MEHP**				
**Endpoint**	**Lowest Effective Dose**	**Exposure**	**Endpoint Time**	**References**
hatching delayed	** 2.8 mg/L	4–76 hpf	76 hpf	[42]
deformity rate increased	** 14 mg/L	4–76 hpf	76 hpf	[42]
body length decreased	** 7.0 mg/L	4–76 hpf	76 hpf	[42]
body length decreased	** 8.4 mg/L	start at embryo stage—3/6 dpf	3/6 dpf	[50]
swim bladder abnormality	200 µg/L	6–96 hpf	96 hpf	[51]
no observed effect concentration (NOEC)	<6.09 mg/L	6–144 hpf	144 hpf	[49]
effective concentration, 10% (EC10)	9.77 mg/L	6–144 hpf	144 hpf	[49]
effective concentration, 50% (EC50)	29.98 mg/L	6–144 hpf	144 hpf	[49]
effective concentration, 100% (EC100)	50.0 mg/L	6–144 hpf	144 hpf	[49]

**Table 3:** Cardiovascular toxicity from acute exposures to (a) DEHP and (b) MEHP on zebrafish. * The concentration was converted from nM or µM using the molar mass of DEHP 390.57 g/mol. ** The concentration was converted from nM or µM using the molar mass of MEHP 278.34 g/mol.

(a) DEHP				
Endpoint	Lowest Effective Dose	Exposure	Endpoint Time	References
heart rate decreased	* 20 mg/L	4–76 hpf	76 hpf	[42]
	120 mg/L	1–72 hpf	72 hpf	[37]
heart rate increased	25, 50 µg/L	3–168 hpf	168 hpf	[38]
pericardial edema	2.5 µg/L	4–72 hpf	72 hpf	[44]
	250 µg/L	1.5–72/96 hpf	72, 96 hpf	[52]
	10 mg/L	72–168 hpf	168 hpf	[42]
apoptosis signal increased in heart region	250 µg/L	1.5–72 hpf	72 hpf	[52]
**(b) MEHP**				
**Endpoint**	**Lowest Effective Dose**	**Exposure**	**Endpoint Time**	**References**
heart rate decreased	** 14 mg/L	4–76 hpf	76 hpf	[42]

**Table 4:** Skeletal toxicity from acute exposure to DEHP on zebrafish embryos.

Endpoint	Lowest Effective Dose	Exposure	Endpoint Time	References
bent spinal curvature	2.5 µg/L	4–72 hpf	72 hpf	[44]
	200 mg/L	1–168 hpf	168 hpf	[37]
spinal dysplasia (cartilage effect)	200 mg/L	1–168 hpf	168 hpf	[37]

**Table 5:** Behavioral toxicity from acute exposures to (a) DEHP and (b) MEHP on zebrafish embryos. * The concentration was converted from nM or µM using the molar mass of DEHP 390.57 g/mol. ** The concentration was converted from nM or µM using the molar mass of MEHP 278.34 g/mol.

(a) DEHP				
Endpoint	Lowest Effective Dose	Exposure	Endpoint Time	References
spontaneous movement decreased (no. of spontaneous movements in 20 s)	50 µg/L	2–24 hpf	24 hpf	[53]
movement inhibition	90 mg/L	1–24 hpf	24 hpf	[37]
locomotor activity inhibited	* 20 mg/L	4–100 hpf	100 hpf	[42]
locomotor activity (dark) (bursting) decreased	5 µg/L	2–120 hpf	120 hpf	[46]
locomotor activity (dark) (cruising) decreased	5 µg/L	2–120 hpf	120 hpf	[46]
locomotor activity (light) (swimming) increased	10 mg/L	2–120 hpf	120 hpf	[46]
locomotor activity (light) (cruising) increased	10 mg/L	2–120 hpf	120 hpf	[46]
locomotor activity (light) (freezeing) increased	1 mg/L	2–120 hpf	120 hpf	[46]
**(b) MEHP**				
**Endpoint**	**Lowest Effective Dose**	**Exposure**	**Endpoint Time**	**References**
locomotor activity inhibited	** 14 mg/L	4–100 hpf	100 hpf	[42]

**Table 6:** Regulation of gene and possible mechanisms from acute exposures to (a) DEHP and (b) MEHP on zebrafish embryos.

(a) DEHP				
Endpoint	Lowest Effective Dose	Exposure	Endpoint Time	References
intense PCNA staining	2.5 µg/L	4–48 hpf	48 hpf	[44]
intense wnt3a staining	2.5 µg/L	4–48 hpf	48 hpf	[45]
intense *β*-catenin staining	2.5 µg/L	4–48 hpf	48 hpf	[46]
*gsk3β* mRNA expression increased	2.5 µg/L	4–72 hpf	72 hpf	[44]
b*mp2* transcript level decreased	50 µg/L	2–96 hpf	96 hpf	[53]
n*gs* transcript level increased	50 µg/L	2–96 hpf	96 hpf	[53]
*co18ala* transcript level increased	50 µg/L	2–96 hpf	96 hpf	[53]
*co18ala* transcript level decreased	250 µg/L	2–96 hpf	96 hpf	[53]
*klh14a* transcript level increased	50 µg/L	2–96 hpf	96 hpf	[53]
smyd2b transcript level increased	50 µg/L	2–96 hpf	96 hpf	[53]
s*pp1* transcript level increased	50 µg/L	2–96 hpf	96 hpf	[53]
s*tac3* transcript level increased	50 µg/L	2–96 hpf	96 hpf	[53]
steroid binding pathway induction *(paqr5b*, *nr1h4*, *fabp10a*)	50 µg/L	1.5–96 hpf	96 hpf	[52]
cyclase activator activity pathway induction (*guca1a*, *guca1d*)	50 µg/L	1.5–96 hpf	96 hpf	[52]
actomyosin structure organization pathway induction (*klhl41a*, *csrp3*, *cnn1b*, *ctnt*)	50 µg/L	1.5–96 hpf	96 hpf	[52]
chemokine receptor binding pathway induction(*ccl27a*, *ccl39.3*)	50 µg/L	1.5–96 hpf	96 hpf	[52]
myofibril assembly pathway induction (*csrp3*, *klhl41a*, *ctnt*)	50 µg/L	1.5–96 hpf	96 hpf	[52]
notochord development pathway induction (*col8a1a*, *ngs*, *fbn2b*)	50 µg/L	1.5–96 hpf	96 hpf	[52]
skeletal system development pathway induction (*spp1*)	50 µg/L	1.5–96 hpf	96 hpf	[52]
heart contraction and heart process pathway induction (*smyd2b,csrp3*)	50 µg/L	1.5–96 hpf	96 hpf	[52]
*nppa* transcript level altered	50 µg/L	1.5–96 hpf	96 hpf	[52]
*My17* transcript level altered	50 µg/L	1.5–96 hpf	96 hpf	[52]
*Tbx5b* transcript level altered	50 µg/L	1.5–96 hpf	96 hpf	[52]
*smyd2b* transcript level altered	50 µg/L	1.5–96 hpf	96 hpf	[52]
*ctnt* transcript level altered	50 µg/L	1.5–96 hpf	96 hpf	[52]
*cmlc1* transcript level altered	50 µg/L	1.5–96 hpf	96 hpf	[52]
hypomethylation (*nppa*, *ctnt*)	50 µg/L	1.5–96 hpf	96 hpf	[52]
hypermethylation (tbx5b)	50 µg/L	1.5–96 hpf	96 hpf	[52]
immune system-related genes (*cci27a*, *tnfaip2b*, *casp3b*, *rnasel3*, *traf3ip2b*, *diexf*, *trpm2*, *park2*, *smyd2b*, *creb312*, *il-1b*, *nfkb1*, *tp63*, *caspa*) altered	50 µg/L	2–96 hpf	96 hpf	[48]
lipid metabolism and skeletal development related genes (*paqr5b*, *nr1h4*, *gc*, *fabp10a*, *esyt3*, *zfos-411a11.2*, *alox5a*, *apoba*, *atp8b1*, *osbp19*, *ch25hl1.2*, *gfpt1*, *klhl41a*, *klhl40b*, *stac3*, *ucmab*, *spp1*, *caspa*, *hapln1b*) altered	50 µg/L	2–96 hpf	96 hpf	[48]
*sp7* transcript level increased	200 mg/L	1–168 hpf	168 hpf	[37]
*runx2b* transcript level increased	200 mg/L	1–168 hpf	168 hpf	[38]
*gpc4a t*ranscript level increased	200 mg/L	1–168 hpf	168 hpf	[39]
*shha* transcript level increased	200 mg/L	1–168 hpf	168 hpf	[40]
*elav13* expression decreased, Fluorescence intensity reduction inTg (HuC:eGFP)	500 µg/L	2–72 hpf	72 hpf	[46]
*ache* transcript level increased	50 µg/L	2–120 hpf	120 hpf	[46]
*th* transcript level decreased	500 µg/L	2–120 hpf	121 hpf	[46]
*pik3r1* mRNA expression increased	400 µg/L	2–168 hpf	168 hpf	[55]
*akt1* mRNA expression increased	400 µg/L	2–168 hpf	168 hpf	[55]
*mtor mRNA* expression increased	400 µg/L	2–168 hpf	168 hpf	[55]
*ps6kb mRNA* expression increased	200 µg/L	2–168 hpf	168 hpf	[55]
AhR activity induction, Fluorescence intensity increased in Tg(cyp1a:gfp)	33 µg/L	2–120 hpf	120 hpf	[55]
Cerg1 level decreased	50 µg/L	2–96 hpf	96 hpf	[48]
Che level decreased	50 µg/L	2–96 hpf	96 hpf	[48]
DG level decreased	50 µg/L	2–96 hpf	96 hpf	[48]
TG level decreased	50 µg/L	2–96 hpf	96 hpf	[48]
FA level decreased	50 µg/L	2–96 hpf	96 hpf	[48]
lipase activity regulation, lipid absorption, lipid catabolism, lipid digestion, lipid metabolism and lipid transport increased	50 µg/L	2–96 hpf	96 hpf	[48]
**(b) MEHP**				
**Endpoint**	**Lowest Effective Dose**	**Exposure**	**Endpoint Time**	**References**
increased vacuolization and type of vacuolization in liver	200 µg/L	6–96 hpf	96 hpf	[51]
lipid accumulation in liver & brain	200 µg/L	6–120 hpf	15 dpf	[51]
*fabp1a1* expression increased	200 µg/L	6–120 hpf	15 dpf	[51]

**Table 7:** Induction of oxidative stress from acute exposures to (a) DEHP and (b) MEHP on zebrafish embryos. * The concentration was converted from nM or µM using the molar mass of DEHP 390.57 g/mol. ** The concentration was converted from nM or µM using the molar mass of MEHP 278.34 g/mol.

(a) DEHP				
Endpoint	Lowest Effective Dose	Exposure	Endpoint Time	References
lipid peroxidation (LPO) increased	2.5 µg/L	start at embryo stage—72 hpf	72 hpf	[40]
glutathione S-transferase (GST) decreased	2.5 µg/L	start at embryo stage—72 hpf	72 hpf	[40]
*CAT* expression increased	50 µg/L	3–96 hpf	96 hpf	[38]
*CuSOD* expression increased	50 µg/L	3–96 hpf	96 hpf	[39]
*MnSOD* expression increased	50 µg/L	3–96 hpf	96 hpf	[40]
ROS generation increased	* 20 mg/L	4–28 hpf	28 hpf	[42]
SOD activity decreased	* 20 mg/L	4–28 hpf	28 hpf	[42]
**(b) MEHP**				
**Endpoint**	**Lowest Effective Dose**	**Exposure**	**Endpoint Time**	**References**
ROS generation increased	** 14 mg/L	4–28 hpf	28 hpf	[42]
SOD activity decreased	** 14 mg/L	4–28 hpf	28 hpf	[42]

**Table 8:** Apoptosis and genotoxicity from acute exposures to (a) DEHP and (b) MEHP on zebrafish embryos. * The concentration was converted from nM or µM using the molar mass of DEHP 390.57 g/mol. ** The concentration was converted from nM or µM using the molar mass of MEHP 278.34 g/mol.

(a) DEHP				
Endpoint	Lowest Effective Dose	Exposure	Endpoint Time	References
apoptosis signal increased	50 µg/L	3–96 hpf	96 hpf	[38]
apoptosis signal increased (heart region)	250µg/L	1.5–72 hpf	72 hpf	[52]
*bax* mRNA expression decreased	50 µg/L	3–96 hpf	96 hpf	[38]
*bax* mRNA expression increased	* 20 mg/L	4–28 hpf	28 hpf	[42]
*bcl2* mRNA expression decreased	* 9.8 mg/L	4–28 hpf	28 hpf	[42]
ctnt transcription level increased	50 µg/L	1.5–96 hpf	96 hpf	[52]
*ctnt* protein level increased	50 µg/L	1.5–96 hpf	96 hpf	[52]
nppa transcription level increased	50 µg/L	1.5–96 hpf	96 hpf	[52]
N*ppa* protein level increased	50 µg/L	1.5–96 hpf	96 hpf	[52]
*cas8* mRNA expression increased	50 µg/L	3–96 hpf	96 hpf	[38]
*cas9* mRNA expression increased	50 µg/L	3–96 hpf	96 hpf	[38]
*pf3* mRNA expression increased	50 µg/L	3–96 hpf	96 hpf	[38]
*ogg1* mRNA expression increased	* 20 mg/L	4–28 hpf	28 hpf	[42]
*parp1* mRNA expression increased	* 3.9 mg/L	4–28 hpf	28 hpf	[42]
*pcna* mRNA expression increased	* 3.9 mg/L	4–28 hpf	28 hpf	[42]
*polb* mRNA expression increased	* 3.9 mg/L	4–28 hpf	28 hpf	[42]
*pold* mRNA expression decreased	* 3.9 mg/L	4–28 hpf	28 hpf	[42]
*fen1* mRNA expression increased	* 20 mg/L	4–28 hpf	28 hpf	[42]
*lig1* mRNA expression increased	* 3.9 mg/L	4–28 hpf	28 hpf	[42]
*c-myc* transcript level increased	2.5 µg/L	start at embryo stage—72 hpf	72 hpf	[40]
DNA breaks increased	5 mg/L	72–168 hpf	168 hpf	[41]
*p53* mRNA expression increased	500 µg/L	72–168 hpf	168 hpf	[41]
*rad51* expression increased	1 mg/L	72–168 hpf	168 hpf	[41]
*xrcc5* expression increased	1 mg/L	72–168 hpf	168 hpf	[41]
**(b) MEHP**				
**Endpoint**	**Lowest Effective Dose**	**Exposure**	**Endpoint Time**	**References**
*bax* mRNA level increased	** 2.8 mg/L	4–28 hpf	28 hpf	[42]
*bcl2* mRNA level decreased	** 14 mg/L	4–28 hpf	28 hpf	[42]
*ogg1* mRNA expression increased	** 2.8 mg/L	4–28 hpf	28 hpf	[42]
*nthl1* mRNA expression increased	** 2.8 mg/L	4–28 hpf	28 hpf	[42]
*apex1* mRNA expression increased	** 7.0 mg/L	4–28 hpf	28 hpf	[42]
*parp1* mRNA expression increased	** 2.8 mg/L	4–28 hpf	28 hpf	[42]
*xrcc1* mRNA expression increased	** 7.0 mg/L	4–28 hpf	28 hpf	[42]
*lig3* mRNA expression increased	** 2.8 mg/L	4–28 hpf	28 hpf	[42]
*ung* mRNA expression increased	** 2.8 mg/L	4–28 hpf	28 hpf	[42]
*pcna* mRNA expression increased	** 2.8 mg/L	4–28 hpf	28 hpf	[42]
*polb* mRNA expression decreased	** 7.0 mg/L	4–28 hpf	28 hpf	[42]
*pold* mRNA expression decreased	** 2.8 mg/L	4–28 hpf	28 hpf	[42]
*fen1* mRNA expression increased	** 7.0 mg/L	4–28 hpf	28 hpf	[42]
*lig1* mRNA expression increased	** 14 mg/L	4–28 hpf	28 hpf	[42]

**Table 9:** Transgenerational effect from acute exposure to MEHP on zebrafish embryos. ** The concentration was converted from nM or µM using the molar mass of MEHP 278.34 g/mol.

Endpoint	Lowest Effective Dose	Exposure	Endpoint Time	References
*dnmt* gene expressions altered (*dnmt1*, *dnmt3aa*, *dnmt3ab*, *dnmt3bb.1*, *dnmt3bb.2*)	F0: ** 8.4 mg/L	F0: start at embryo stage—6 dpf	F0 6 dpf	[50]
global demethylation in liver	F0: ** 8.4 mg/L	F0: start at embryo stage—6 dpf	F0 adult female	[50]
global hmC level decreased	F0: ** 8.4 mg/L	F0: start at embryo stage—6 dpf	F0 6 dpf	[50]
global hmC level decreased in brain tissue	F0: ** 8.4 mg/L	F0: start at embryo stage—6 dpf	F0 adult male	[50]
average methylation increased at zfCNEs	F0: ** 8.4 mg/L	F0: start at embryo stage—6 dpf	F0 6 dpf	[50]
adipogenesis pathway increased	F0: ** 8.4 mg/L	F0: start at embryo stage—6 dpf	F0 6 dpf	[50]
transgenerational effect: DNA methylation at *cbfa2t2* locus and specific CpG sites among F0, F1 and F2 generations	F0: ** 8.4 mg/L	F0: start at embryo stage—6 dpf	F0 6 dpf, F1 6 dpf, F2 6 dpf, F0 15 dpf and F0 adult sperm	[50]

**Table 10:** Endocrine disruption from acute exposures to (a) DEHP and (b) MEHP in zebrafish embryos. ** The concentration was converted from nM or µM using the molar mass of MEHP 278.34 g/mol.

(a) DEHP				
Endpoint	Lowest Effective Dose	Exposure	Endpoint Time	References
triiodothyronine (T3) level increased	400 µg/L	2–168 hpf	168 hpf	[59]
triiodothyronine (T3) level increased	250 µg/L	2–96 hpf	96 hpf	[48]
thyroxinem (T4) level increased	400 µg/L	2–168 hpf	168 hpf	[59]
thyroxinem (T4) level increased	250 µg/L	2–96 hpf	96 hpf	[48]
estrogen receptor alpha *(erα)* *mRNA* increased	50 µg/L	3–96 hpf	96 hpf	[38]
estrogen receptor alpha *(erα)* *mRNA* increased	250 µg/L	2–96 hpf	96 hpf	[48]
estrogen receptor alpha *(ERα)* gene expression increased	100 µg/L	start at embryo stage—168 hpf	168 hpf	[60]
estrogen receptor alpha *(ERα*) protein level increased	250 µg/L	2–96 hpf	96 hpf	[48]
estrogen receptor alpha *(erα)* transcript level decreased	10 µg/L	6–168 hpf	168 hpf	[47]
estrogen receptor beta *(erβ)* mRNA increased	50 µg/L	3–96 hpf	96 hpf	[38]
cytochrome P450 aromatase *(cyp19a)* expression increased	25 µg/L	3–96 hpf	96 hpf	[38]
cytochrome P450 aromatase *(Cyp19b)* gene expression increased	100 µg/L	start at embryo stage—168 hpf	168 hpf	[60]
cytochrome P450 aromatase *(cyp19a1b)* transcript level increased	10 µg/L	6–168 hpf	168 hpf	[47]
vitellogenin *(vtg)* mRNA expression increased	25 µg/L	3–96 hpf	96 hpf	[38]
vitellogenin *(Vtg)* gene expression increased	100 µg/L	start at embryo stage—168 hpf	168 hpf	[60]
vitellogenin *(vtg1)* transcript level increased	100 µg/L	6–168 hpf	168 hpf	[47]
thyroid stimulating hormone *(tshβ)* mRNA expression increased	100 µg/L	2–168 hpf	168 hpf	[59]
corticotrophin releasing hormone *(crh)* mRNA expression increased	200 µg/L	2–168 hpf	168 hpf	[59]
NK2 homeobox 1 *(nkx2.1)* mRNA expression increased	200 µg/L	2–168 hpf	168 hpf	[59]
thyroglobulin *(tg)* mRNA expression increased	400 µg/L	2–168 hpf	168 hpf	[59]
uridinediphosphate-glucuronosyl-transferase *(ugtlab)* mRNA expression decreased	200 µg/L	2–168 hpf	168 hpf	[59]
iodothyronine deiodinase *(dio2)* mRNA expression increased	400 µg/L	2–168 hpf	168 hpf	[59]
transthyretin *(ttr)* mRNA expression increased	200 µg/L	2–168 hpf	168 hpf	[59]
androgen receptor *(ar)* mRNA expression increased	50 µg/L	3–96 hpf	96 hpf	[38]
17*β*estradiol level increased	250 µg/L	2–96 hpf	96 hpf	[48]
cancer cell migration (from yolk to gut and tail)	400 µg/L	72–78 hpf	78 hpf	[55]
**(b) MEHP**				
**Endpoint**	**Lowest Effective Dose**	**Exposure**	**Endpoint Time**	**References**
triiodothyronine (T3) level increased	200 µg/L	2–168 hpf	168 hpf	[62]
thyroxinem (T4) level decreased	200 µg/L	2–168 hpf	168 hpf	[62]
thyroid stimulating hormone (TSH*β*) transcription increased	40 µg/L	2–168 hpf	168 hpf	[62]
NK2 Homeobox 1 *(Nkx2.1)* transcription increased	8 µg/L	2–168 hpf	168 hpf	[62]
thyroglobulin *(TG)* transcription increased	200 µg/L	2–168 hpf	168 hpf	[62]
UDP-glucuronosyltransferases *(UGT1ab)* transcription increased	40 µg/L	2–168 hpf	168 hpf	[62]
iodothyronine deiodinase *(Dio1)* transcription increased	8 µg/L	2–168 hpf	168 hpf	[62]
iodothyronine deiodinase *(Dio2)* transcription increased	40 µg/L	2–168 hpf	168 hpf	[62]
transthyretin *(TTR)* transcription decreased	200 µg/L	2–168 hpf	168 hpf	[62]
paired box 8 *(Pax8)* transcription increased	8 µg/L	2–168 hpf	168 hpf	[62]
sodium/iodide symporter *(NIS)* transcription increased	40 µg/L	2–168 hpf	168 hpf	[62]
GSH disruption in body, heart, brain ventricle and somite 12 (MCB fluorescence decreased)	200 µg/L	3–48/72 hpf	48/72 hpf	[63]
hypomorphic islets (*β*-cell cluster area decreased)(Tg (ins:GFP))	** 195 µg/L	3–48 hpf	48 hpf	[65]
pancreatic islet variants and defects decreased	** 195 µg/L	3–48 hpf	48 hpf	[65]
hypomorphic islets (*β*-cell cluster area decreased)(Tg (ins:GFP))	200 µg/L	3–48/72/96/168 hpf	48/72/96/168 hpf	[64]
hypomorphic islets (*α*-cell cluster area decreased) (Tg (gcga:GFP))	200 µg/L	3–48/72/96/168 hpf	48/72/96/168 hpf	[64]
frequency of pancreatic islet morphology variants increased	200 µg/L	3–48/72/96/168 hpf	48/72/96/168 hpf	[64]
pancreatic structure altered in wt zebrafish embryo	200 µg/L	3–48/72/96 hpf	48/72/96 hpf	[64]
pancreatic structure altered in m zebrafish embryo	200 µg/L	3–48/72/96 hpf	48/72/96 hpf	[64]
preproinsulin a *(insa)* expression decreased	200 µg/L	3–96 hpf	96 hpf	[64]
somatostatin 2 *(sst2)* expression decreased	200 µg/L	3–96 hpf	96 hpf	[64]
pancreasspecifictranscription factor 1a *(ptf1a)* expression decreased	200 µg/L	3–96 hpf	96 hpf	[64]
glutathione-disulfide reductase *(gsr)* expression increased	200 µg/L	3–96 hpf	96 hpf	[64]
glutathione S-transferase pi 1 *(gstp)* expression decreased	200 µg/L	3–96 hpf	96 hpf	[64]

**Table 11:** Effects from chronic exposure to DEHP on zebrafish embryos.

Endpoint	Lowest Effective Dose	Exposure	Endpoint Time	References
mortality rate increased	0.5 µg/L	27 hpf—1 month	1 month	[39]
body length increased (male)	33 µg/L	start at embryo stage—3.5 month	3.5 month	[66]
body length decreased (male)	0.5 µg/L	27 hpf—6 month	6 month	[39]
body length increased (female)	10 µg/L	start at embryo stage—3.5 month	3.5 month	[66]
body length decreased (female)	0.5 µg/L	27 hpf—6 month	6 month	[39]
body weight increased (male)	10 µg/L	start at embryo stage—3.5 month	3.5 month	[66]
body weight decreased (male)	0.5 µg/L	27 hpf—6 month	6 month	[39]
body weight increased (female)	10 µg/L	start at embryo stage—3.5 month	3.5 month	[66]
body weight decreased (female)	0.5 µg/L	27 hpf—6 month	6 month	[39]
intestinal microbiota alteration (female)	100 µg/L	start at embryo stage—3.5 month	3.5 month	[66]
intestinal microbiota alteration (male)	100 µg/L	start at embryo stage—3.5 month	3.5 month	[66]
intestinal bacteria alteration (female)	100 µg/L	start at embryo stage—3.5 month	3.5 month	[66]
intestinal bacteria alteration (male)	100 µg/L	start at embryo stage—3.5 month	3.5 month	[66]
villus width decreased (male)	100 µg/L	start at embryo stage—3.5 month	3.5 month	[66]
tunica muscularis thickness decreased (female)	100 µg/L	start at embryo stage—3.5 month	3.5 month	[66]
goblet cells per villus decreased (female)	100 µg/L	start at embryo stage—3.5 month	3.5 month	[66]
*tlr-5* mRNA expression decreased (female)	100 µg/L	start at embryo stage—3.5 month	3.5 month	[66]
*il-1β* mRNA expression decreased (female)	10 µg/L	start at embryo stage—3.5 month	3.5 month	[66]
*il-8* mRNA expression increased (male)	10 µg/L	start at embryo stage—3.5 month	3.5 month	[66]
*il-8* mRNA expression increased (female)	10 µg/L	start at embryo stage—3.5 month	3.5 month	[66]
TG content increased (male)	33 µg/L	start at embryo stage—3.5 month	3.5 month	[66]
TG content increased (female)	10 µg/L	start at embryo stage—3.5 month	3.5 month	[66]
PY content increased (male)	10 µg/L	start at embryo stage—3.5 month	3.5 month	[66]
PY content increased (female)	33 µg/L	start at embryo stage—3.5 month	3.5 month	[66]
FA content increased (male)	10 µg/L	start at embryo stage—3.5 month	3.5 month	[66]
Glu content increased (female)	10 µg/L	start at embryo stage—3.5 month	3.5 month	[66]
conditional factor increased (male)	33 µg/L	start at embryo stage—3.5 month	3.5 month	[66]
conditional factor increased (female)	10 µg/L	start at embryo stage—3.5 month	3.5 month	[66]
Gonad somatic index (GSI) decreased (male)	0.5 µg/L	27 hpf—6 month	6 month	[39]
Gonad somatic index (GSI) decreased (female)	0.5 µg/L	27 hpf—6 month	6 month	[39]
egg production decreased	0.5 µg/L	27 hpf—6 month	6 month	[39]
non-fertilization rate increased	0.5 µg/L	27 hpf—6 month	6 month	[39]
spermatogonia and spermatocyte decreased (male)	0.5 µg/L	27 hpf—6 month	6 month	[39]
tubules distruption (male)	0.5 µg/L	27 hpf—6 month	6 month	[39]
ovary development inhibition (female)	0.5 µg/L	27 hpf—6 month	6 month	[39]
number of vitellogenic oocytes decreased (female)	0.5 µg/L	27 hpf—6 month	6 month	[39]
